# Reducing Test Anxiety: A Randomized Controlled Pilot Study of Evening Bright Light Exposure in University Students

**DOI:** 10.1155/da/1422406

**Published:** 2025-08-26

**Authors:** Maximilian Dick, Helmut K. Lackner, Elisabeth M. Weiss, Markus Canazei

**Affiliations:** ^1^Department of Psychology, University of Innsbruck, Innsbruck, Austria; ^2^Research and Development Department, Bartenbach GmbH, Wattens, Austria; ^3^Division of Physiology, Otto Loewi Research Center, Medical University of Graz, Graz, Austria

**Keywords:** bright evening light exposure, cognitive performance, dim evening light exposure, heart rate variability, sleep, test anxiety

## Abstract

**Background:** University students often experience high levels of stress and anxiety during exam periods, adversely affecting their well-being and academic performance. This study investigated the short-term effects of evening bright light (BL) exposure on several psychophysiological stress measures during exam preparation.

**Methods:** In this preregistered randomized controlled pilot study, 35 university students were assigned either to an intervention group exposed to BL (1500 lx, 4000 K; *n* = 18) or a control light (CL) group with standard lighting (100 lx, 3000 K; *n* = 17) for 4 h over five consecutive evenings. Outcomes included questionnaires (test anxiety, general anxiety, psychological distress), cognitive performance (2-back, go-/no-go task [GNT]), physiological stress (heart rate variability [HRV]), and subjective and objective sleep quality measures (actigraphy).

**Results:** The BL group showed significant reductions in test anxiety by the last evening. Both groups improved in working memory performance over time. HRV analysis revealed mixed results, with some indications of reduced stress in the BL group on the first day. No adverse effects of evening BL were found on sleep parameters, and participants reported significantly higher satisfaction with the BL exposure.

**Conclusions:** Evening BL exposure during exam preparation may help reduce test anxiety without significantly disrupting sleep. Although cognitive performance effects were limited, the perceived usefulness suggests that BL could be a well-accepted supportive measure for students during stressful academic periods. Further research is needed to optimize light-based interventions for student well-being.

## 1. Introduction

### 1.1. Stress and Anxiety in University Students

University students frequently encounter significant stress levels triggered by various factors. Academic stressors such as regular performance reviews, work overload, a competitive environment, and future concerns, along with nonacademic factors like adjusting to new living environments, developing personal identity, adapting to new social settings, and managing finances, contribute to student stress [1, 2]. Test anxiety, a specific form of anxiety characterized by excessive worry and fear about academic performance, is particularly prevalent among university students, with studies reporting prevalence rates ranging from 39% up to 56%, depending on the measurement tools and populations studied [3–5]. Academic stress is closely linked to mental well-being issues such as anxiety, depression, and suicidal ideation, especially in medical students [6, 7]. The COVID-19 pandemic and subsequent lockdowns intensified existing stress and related mental health problems like depression and anxiety in students [6, 8–13]. Over time, chronic stress and anxiety can lead to reduced sleep quality [14–16], decreased academic performance [17], and severe mental health conditions such as anxiety disorders, affective disorders, and substance abuse [18].

### 1.2. Current Treatments and Their Limitations

Current approaches to treating elevated stress, anxiety, and depression in university students include a variety of interventions, each with its own advantages and limitations [19]. Despite the availability and effectiveness of these treatments, many students do not seek help or lack access to it [18, 20].

Mindfulness-based interventions, such as progressive muscle relaxation, breathing exercises, or mindfulness-based stress reduction, have gained popularity due to their high accessibility and effectiveness in reducing stress and anxiety [21–23]. However, these interventions require continuous practice for optimal results, which can be challenging, especially for more severely affected students. Cognitive-behavioral therapy is another effective treatment option [24–26], offering good long-term outcomes but often limited by high costs and long waiting times for treatment [27]. In addition, symptom improvement typically takes several weeks, potentially impacting patient compliance. Psychotropic medications (e.g., selective serotonin reuptake inhibitors or benzodiazepines) are widely prescribed and can significantly reduce symptoms but may cause adverse effects. Benzodiazepines, in particular, present a serious risk of dependency with long-term use and should not be considered a first-line treatment [28].

### 1.3. Bright Light (BL) Therapy

A nonpharmacological treatment option for depression symptoms is morning BL exposure, with intensities up to 10,000 lx [29]. BL therapy can also be included as adjunctive treatment into an existing treatment plan. The beneficial effects of BL therapy are well documented, particularly for treating seasonal affective disorder [30–33]. Additionally, there is growing evidence supporting its effectiveness for nonseasonal depression, as highlighted in recent reviews and meta-analyses [30, 33–37]. Beyond affective disorders, BL therapy has shown benefits in conditions such as perinatal depression [38], bipolar disorder [33, 39, 40], eating disorders [41], dementia [42, 43], borderline personality disorder [44], schizophrenia [45], combat-related PTSD [46], ADHD [47], cancer-related fatigue [48], poststroke insomnia [49], and burnout symptoms [50]. However, research specifically examining the effects of BL on anxiety disorders remains limited and inconsistent. One study found a small effect in individuals with low anxiety levels [51], while another found no effect in those with high anxiety levels [52]. Nonetheless, BL therapy has been shown to reduce anxiety symptoms in patients with nonpsychiatric disorders such as focal epilepsy [53] and Parkinson's disease [54].

### 1.4. Some Nonclinical Effects of BL Exposure

#### 1.4.1. Acute Cognitive Effects

Apart from conventional BL therapy used in clinical populations, systematic reviews have increasingly shown evidence of the acute alerting effects of BL exposure in healthy individuals when applied at different times of the day [55–57]. Recent meta-analyses have found that increased illuminance improves both subjective and objective alertness measures, and higher correlated color temperature (CCT) enhances these measures during both daytime and nighttime exposure [58, 59]. However, findings related to more complex cognitive functions, such as working memory, are inconclusive. While higher illuminance and CCT tend to improve cognitive performance (including sustained and selective attention, memory, executive functions, and processing speed), the relationship depends on the specific cognitive task and its difficulty. Overall, further research is needed to clarify these effects [59–61].

#### 1.4.2. Acute and Transient Effects on Sleep

Research has demonstrated that daytime BL exposure positively affects sleep quality [62], while evening BL exposure can have adverse effects, such as increasing sleep onset latency, enhancing slow wave sleep, and reducing sleep efficiency, as shown in recent polysomnographic studies [63]. Interestingly, early evening light exposure (6:30 p.m. to 9 p.m.), in contrast to late evening light exposure (10:30 p.m. to 11:30 p.m.), may not exert alerting effects on humans [64].

Sleep is also often assessed using wrist actimetry, a validated method for evaluating sleep parameters when polysomnography is unavailable or impractical [65]. A recent review and meta-analysis using actigraphy found no evidence of light intervention effects on sleep parameters in dementia patients [66].

### 1.5. Adverse Effects of Evening BL Exposure

The term “evening” is broadly used in many studies without a clear, standardized definition. A potential physiological indicator is the onset of melatonin secretion for nocturnal sleep, known as dim light melatonin onset, which typically occurs around 2–3 h before usual bedtime and could be described as “evening.” However, this timing varies individually and requires specialized equipment to measure [67]. As previously mentioned, evening light exposure delays melatonin secretion, adversely affecting sleep. This effect is influenced by the melanopic equivalent daylight illuminance (EDI) of the light exposure [63]. Over extended periods and in severe cases, such as with night shift workers, this can lead to circadian disruption, a known risk factor for numerous adverse health effects, including metabolic disorders, cardiovascular diseases, neurological disorders, gastrointestinal ulcers, and various cancers [68, 69].

Similarly, nighttime light exposure, defined by Burns et al. [70] as exposure after 12:30 a.m., is linked to an increased risk of mental disorders such as depression, generalized anxiety disorder, PTSD, and psychosis. In contrast, daytime light exposure is associated with a reduced risk of these conditions [70]. A recent review confirmed these results [71].

### 1.6. Hypotheses

Given the prevalence of stress and anxiety among university students and the potential benefits of BL interventions, it is important to explore the possible positive effects of BL exposure during intense study periods. University students commonly go to bed late, with most retiring after 11 p.m. [72, 73], and a significant portion uses this time to study for exams, with 69% studying in the evening and 20% late at night [74]. Therefore, we designed our study to take place in the evening while monitoring for any adverse effects of the light interventions.

Our study aims to investigate both the potential benefits and possible detrimental effects of a 5-day daily evening BL intervention for students experiencing increased test anxiety during exam preparation periods. The use of BL intervention for subsyndromal test anxiety is a novel approach in our research. We categorized the outcome parameters into three distinct groups according to the primary and secondary objectives of our study: psychological/psychiatric measures (test anxiety, general anxiety, and general psychological distress) were categorized as primary outcomes; cognitive performance (working memory and response inhibition), sleep variables (presleep arousal, self-reported sleep quality, and actigraphically measured sleep quality), and physiological stress markers (heart rate and heart rate variability [HRV]) were categorized as secondary outcomes; perceived usefulness of the light intervention and academic performance were categorized as tertiary outcomes.

Based on existing literature, we hypothesized that daily evening exposure to BL compared to a control light (CL) group simulating common home workplace lighting for 4 h over 5 consecutive weekdays during an intensive exam preparation phase would reduce test anxiety, general anxiety, and psychological distress, while improving working memory and response inhibition performance. Furthermore, we expected no significant differences in presleep arousal, self-reported sleep quality, and actigraphically measured sleep quality but anticipated improved HRV parameters during cognitive tasks and sleep. Lastly, we expected higher perceived usefulness of the brightly lit study environment, along with better academic performance.

## 2. Materials and Methods

### 2.1. Measures

#### 2.1.1. Questionnaires

Several questionnaire batteries were utilized in this study: a screening questionnaire to assess participant eligibility, a characterization questionnaire administered 1 week prior to participation, outcome questionnaires recorded at three time points, daily sleep assessments, and a follow-up questionnaire conducted three to 4 weeks postparticipation, after participants received their exam grades. Detailed descriptions of the questionnaires are provided below and summarized in Table S1.

The screening questionnaire included measures of test anxiety (Test Anxiety Inventory; TAI-G; [75, 76]), chronotype (Munich ChronoType Questionnaire; μMCTQ; [77]), and depressive symptoms and suicidal ideation (Patient Health Questionnaire; PHQ-9; [78]; Beck Depression Inventory II; BDI-II; [79]).

The characterization questionnaire comprised the General Depression Scale (ADS-L; [80]) to evaluate depressive symptoms, the Personal Inventory for Depression and SAD (PIDS-SA; [81]) to assess potential seasonal affective disorder, the State–Trait Anxiety Inventory-Trait (STAI-T; [82–84]) to measure trait anxiety, the Pittsburgh Sleep Quality Index (PSQI; [85, 86]) to evaluate subjective sleep quality, and the Perceived Stress Scale (PSS-10; [87, 88]) to assess perceived stress levels.

As outcome measures, we recorded test anxiety using the Test Anxiety Questionnaire for Students (PAF-S; [89]), psychological distress with the Brief Symptom Inventory (BSI-18; [90]), and state anxiety using the State–Trait Anxiety Inventory-State (STAI-S; [82–84]). Since norm data for the PAF-S are available, we converted raw scores into age- and gender-corrected *T* values. These questionnaires were presented on the mornings of the first day (M1), third day (M2), and sixth day (M3). At M1, we included a question about the expected usefulness of the study workplace (“*I think that the study workplace with the luminaire can help me to study during an intensive exam preparation period*.”; rated on a 5-point Likert scale). At M3, we added three questions assessing the perceived usefulness (“*I would use the study workplace with the luminaire again when I am in an intensive exam preparation period*.”; “*I would recommend the study workplace with the luminaire to friends who are in an intensive exam preparation period*.”; “*I think that the study workplace with the luminaire helped me to study during an intensive exam preparation period*.”; each rated on a 5-point Likert scale).

Daily sleep assessments included the Pre-Sleep Arousal Scale (PSAS; [91, 92]), which measures physical and cognitive arousal and was administered across all five workdays immediately before sleep. Upon awakening each workday, participants rated their sleep quality using a single-item measure (“*Please rate the quality of your sleep last night*.”) on a five-point Likert scale and logged their bedtimes (“*What time did you go to bed last night?*” and “*What time did you get out of bed this morning?*”).

Finally, in the follow-up questionnaire, approximately 2–3 weeks after participation, we recorded the grade participants received for the exam they studied for during the study.

Daily assessments (PSAS, sleep quality, bedtime logging) were collected via smartphone app (www.expiwell.com, ExpiWell LLC, West Lafayette, USA), while all other questionnaires were administered online using LimeSurvey (www.limesurvey.org, LimeSurvey GmbH, Hamburg, Germany).

#### 2.1.2. Cognitive Performance Tests

Working memory capacity was assessed using a dual 2-back task [93], a method frequently employed in light impact research (e.g., [94–99]). In this study, visual and auditory stimuli were presented simultaneously: blue squares appeared at eight random locations on a black computer screen for the visual component, while eight letters were spoken in random order through headphones for the auditory component. This multimodal presentation ensured participants maintained visual focus on the screen, thereby keeping corneal light exposure at eye level stable.

Participants were instructed to press designated response keys if a stimulus matched the one presented two trials earlier for each modality separately. No response was required for nonmatching stimuli. After 12 training trials, which were excluded from data analysis, the test comprising 66 trials (33 visual and 33 auditory) began and lasted for 5 min. Visual stimuli were presented for 500 ms, with a 2500 ms response window for both modalities.

Performance was quantified by calculating the average of correct visual and auditory hits (total hits minus false alarms for each modality, divided by the total number of experimental blocks). This measure accounts for both sensory modalities and allows comparisons across different experimental designs, as suggested by the original authors of the dual *n*-back task [93].

Response inhibition, a key component of executive function, was assessed using a visual go-/no-go task (GNT) [100]. This task has also been previously used in light impact research [101, 102]. In the GNT, 128 rectangles were presented over a 5-min period, alternating between green target stimuli and blue distractor stimuli in vertical and horizontal orientations. Participants were instructed to press the spacebar for green rectangles (go) and withhold responses for blue rectangles (no-go). Each trial consisted of an 800 ms white fixation cross, followed by a 300 ms blank black screen, a 1000 ms stimulus presentation, and another 300 ms blank black screen before the next trial began. Only responses with reaction times between 100 and 1000 ms were counted as correct. The primary outcome measure was reaction speed for correct responses, calculated as inverted reaction time. The secondary outcome was the number of commission errors (responses to blue rectangles).

Both cognitive performance tests were administered using Inquisit Lab (www.millisecond.com, Millisecond Software LLC, Seattle, USA) and presented on a 32” monitor (Philips Momentum 326M6VJRMB, Koninklijke Philips, Amsterdam, Netherlands) with a black background. The monitor's luminance was set at 70 cd/m^2^ with a gray desktop background.

#### 2.1.3. Actigraphy and HRV

ActiGraph wGT3X-BT devices (ActiGraph LLC, Pensacola, USA) were used in this study. Participants wore these devices continuously for 6 days (from Monday evening until Saturday morning) on their nondominant wrists, removing them only while showering. The sampling frequency for three-dimensional accelerometer data was set at 100 Hz. Raw data were processed using ActiLife software (version 6.13.5; ActiGraph LLC, Pensacola, USA) and aggregated into 1-min epochs. Sleep quality parameters, including sleep efficiency, total sleep time, wake after sleep onset, number of awakenings, and sleep onset latency, were calculated using the Cole–Kripke algorithm [103].

Given the documented effects of light interventions on the autonomic nervous system as measured by heart rate or HRV parameters [55], we monitored for potential physiological adverse effects of evening BL exposure. Electrocardiogram (ECG) and 3D acceleration monitoring were conducted using portable Bittium Faros 180 devices (Bittium Corporation, Oulu, Finland), worn twice for 24 h (from the first evening until the second evening and from the fifth evening until the sixth evening). The devices were set to single-channel ECG with an Einthoven Lead II set-up, a sampling rate of 1 kHz, and a 3D acceleration sensor sampling rate of 100 Hz. Raw data were converted from EDF to MATLAB data format for further analysis. To ensure ECG data quality and calculate interbeat interval time series, we applied semi-automatic artifact-handling software. This software identifies ectopic beats based on QRS complex patterns and timing within the ECG, applies physiological limits on an individual age-depending basis, and considers the maximum percentage change relative to the signal's standard deviation [104]. ECG data during sleep were divided into restful and restless phases using ECG-derived respiration, as detailed in Hackl-Wimmer et al. [105, 106].

All HRV variables were calculated following the recommended guidelines [107]. For these calculations, 120-s interval phases were used to ensure signal stability and stationarity, considering that segments should be at least 10 times the wavelength of the lower frequency bound of the component being investigated.

Time-domain HRV parameters included SDNN (standard deviation of the regular R–R intervals) and RMSSD (square root of the mean squared differences of successive R–R intervals). RMSSD is more influenced by vagal tone (parasympathetic activity), while SDNN reflects both sympathetic and parasympathetic activity [108, 109].

For frequency-domain HRV variables, power spectral density (PSD) estimates were calculated from R–R intervals using Burg's method (model order 24) after detrending (2nd-order). Low frequency (LF) was defined as 0.04–0.15 Hz, and high frequency (HF) as 0.15–0.40 Hz. Due to skewed distribution in frequency domain variables, a natural logarithmic transformation was applied, represented in ln (LF/HF).

### 2.2. Study Protocol

The study used a two-factor mixed repeated measures design. The between-subjects factor was the light intervention (two levels: BL and CL), and the within-subjects factor was the time of assessment (three levels). Questionnaire data were collected on the first, third, and sixth mornings, while cognitive performance was assessed on the first, third, and fifth evenings. For sleep data (self-reported sleep quality and actigraphically measured sleep parameters), there were five levels (five nights), and for HRV during cognitive tasks and sleep, there were two levels (two 24-h periods).

Participants were recruited from the Medical University of Innsbruck and the University of Innsbruck, focusing on students studying medicine, law, or STEM subjects. These areas were chosen because they involve major exams requiring extensive preparation (minimum of 10 ECTS points per exam to be eligible for study participation), leading to prolonged exam stress.

Students were contacted via the study program mailing list and directed to an online screening questionnaire. This collected data on test anxiety (TAI-G), chronotype (μMCTQ), depression (PHQ-9), suicidal ideation (Item 9 from the BDI-II), as well as any pre-existing psychiatric, internal, or neurological disorders, current medication, eye diseases, and use of visual aids. Sociodemographic information (gender, age, study program) and details about their next major exam were also gathered. Participants were told at study start that the study investigated the effects of an “innovative workplace lighting concept.”

Students with TAI-G scores below 60, PHQ-9 scores above 14, or item 9 scores from the BDI-II (suicidal ideation) above 1 were excluded. Those with pre-existing conditions, current medication (except birth control), or eye diseases were also excluded. Participation was scheduled between 4 weeks to 1 week before their exams.

One week before participation, subjects completed a characterization questionnaire via an online survey, covering depression (ADS-L), seasonal affective disorder (PIDS-SA), trait anxiety (STAI-T), sleep disturbances (PSQI), and perceived stress (PSS-10).

On the weekend before participating in the study, participants were asked to practice the two cognitive tests (2-back task, GNT) twice each on Saturday and Sunday. This practice aimed to minimize expected learning effects during the study due to repeated administration, which are well documented for *n*-back tasks [93, 110].

On the morning of the first study day (Monday; M1), participants completed an online questionnaire assessing test anxiety (PAF-S), depression, anxiety and somatization (BSI-18), state anxiety (STAI-S), their expected exam grade, current overall mean grade, and the anticipated usefulness of the study workplace luminaire (participants had not yet seen the luminaire at this point). In the evening, participants arrived at the laboratory around 5:30 p.m. They were fitted with a wrist actimeter and three chest electrodes for ECG recording. They then completed the first run of the two cognitive tests. Afterward, workplace lighting (BL or CL, with a maximum of four subjects per group) was activated, and participants were instructed to study for their exam using the workplace monitor. After 3 h, they completed the second run of cognitive tests and had 1 h left for studying. After 4 h, the light intervention ended, and participants completed the third run of cognitive tests under ambient lighting provided by free-standing luminaires. Participants were instructed to maintain their usual sleep schedule over the next 5 study days and then went home. Immediately before going to bed, they completed the presleep arousal questionnaire.

On the second day (Tuesday), after awakening, participants rated their subjective sleep quality (one item on a 5-point scale) and recorded their sleep and wake times. In the evening, around 6:00 p.m., they returned to the laboratory, where ECG electrodes and recording devices were removed. At 6:30 p.m., workplace lighting was activated again, allowing subjects to study uninterrupted for 4 h under the specific light intervention.

**Figure 1:**
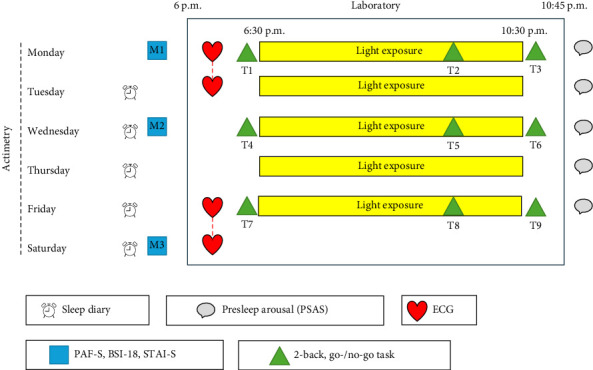
Study protocol.

On the morning of the third day (Wednesday; M2), participants completed an online questionnaire with PAF-S, BSI-18, and STAI-S. The Wednesday evening laboratory session mirrored that of Monday evening, except no ECG was recorded. The fourth day (Thursday) followed the same protocol as Tuesday. The fifth study day (Friday) was similar to Monday but without completing an online questionnaire until Saturday morning (M3). On Saturday, participants completed the questionnaire in the morning and returned actimeters and ECG recording devices in the evening. The weekly study protocol is visualized in Figure 1. About 2–3 weeks after study completion, participants received a follow-up questionnaire to report their exam grades.

Data collection occurred over two winter periods: October 2021 to March 2022 and November 2022 to March 2023 in Innsbruck, Austria. The light intervention was conducted from 6:30 p.m. to 10:30 p.m., fixed at exactly 4 h each evening for five consecutive days. The study protocol received approval from the Ethics Review Board of the University of Innsbruck (No. 32/2020). All participants provided written informed consent before participating and received financial compensation.

The study was preregistered on the Open Science Framework, with the protocol available at https://doi.org/10.17605/OSF.IO/P3VQR. As outlined in the preregistration protocol, an interim analysis of sleep data was conducted during the Christmas break of the first winter period to assess for potential adverse effects of evening BL exposure. Since no detrimental effects on subjective and objective sleep quality were observed (as measured by questionnaires, wrist actimetry, and nighttime ECG recordings), the study protocol remained unchanged.

The target sample size was determined based on previous literature on BL effects on anxiety since no previous studies investigated specific test anxiety. We also conducted a power analysis of the study by Youngstedt and Kripke [51], who first found an anxiolytic effect of BL exposure. The power analysis indicated a required sample size of *n* = 82, and sample sizes in the previous literature ranged between 10 and 144 [46, 51–53, 111–113]. Considering both the power analysis results and precedent sample sizes, we set a target sample size of *n* = 100 for this study.

### 2.3. Workplace Luminaire

The study utilized a research prototype workplace luminaire developed by Bartenbach GmbH (Aldrans, Austria). This lighting system incorporated 72 warm-white LEDs with a CCT of 2200 K and 72 cold-white LEDs with a color temperature of 5300 K (LEDs were Lumileds Luxeon Z-ES, Lumileds Holding B. V., Schiphol, Netherlands). The luminaire operated at a maximum power consumption of 288 W, achieving an average efficacy of 101 lm/W. The LED light sources were completely shielded from direct view using specific reflector technology. Additionally, the light control system allowed for independent illumination of the back wall and desk surface, providing high flexibility in creating the two workplace lighting interventions.

### 2.4. Workplace Lighting Conditions and Room Setting

Two light interventions were implemented in this study. In the CL group, participants were exposed to 94 ± 5 lx at 2838 ± 112 K at eye level, resulting in a melanopic EDI of 50 ± 5 lx at eye level. Under CL conditions, only the desk surface was illuminated. In the BL condition, participants were exposed to 1531 ± 22 lx at 3868 ± 43 K at eye level, resulting in a melanopic EDI of 1017 ± 23 lx at eye level. Under BL conditions, both the back wall and the desk surface were illuminated.

These melanopic EDI levels are relatively high for evening light exposure: a recent expert consensus recommends a maximum melanopic EDI of 10 lx at eye level in the evening, at least 3 h before bedtime, while light exposure during sleep should be minimized with a maximum melanopic EDI of 1 lx. During the day, a minimum melanopic EDI of 250 lx should be achieved [114].

Actual measured illuminances and CCTs varied slightly among the four workplaces (Table S11). Illuminance and spectral measurements were taken with a Gossen MAVOSPEC BASE (https://gossen-photo.de/en/mavospec-base/, GOSSEN Foto- und Lichtmesstechnik GmbH, Hamburg, Germany). Full spectral measurement data for each workplace and light intervention are available as .csv files in Supporting Informations 1 and 2. All calculations were performed using the open-source tool luox (https://luox.app/) [115].

**Figure 2:**
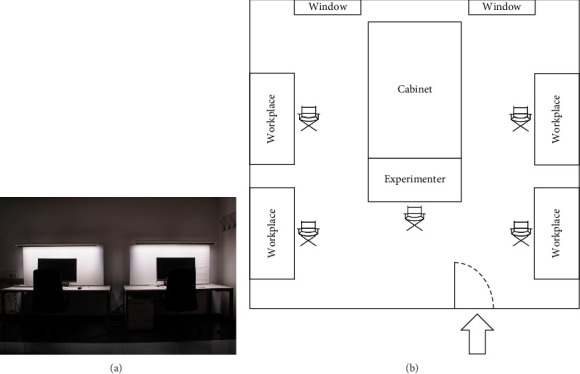
(a) Two workplaces set to the BL condition; (b) schematic representation of the room layout.

To eliminate residual daylight and street lighting from entering the study room, window blinds were fully closed (Figure 2). Ambient lighting before and after the 4-h light exposure was provided by free-standing luminaires, which emitted light at a CCT of 4000 K, delivering an illuminance of ~57 lx at desk level and 11 lx at eye level. During the 4-h light exposure period, these luminaires were turned off, and all workplace luminaires were set to the same light intervention condition. Although ambient air temperature was not monitored throughout the study, it was measured in an internal pilot study and ranged between 22 and 24°C.

### 2.5. Participants

**Figure 3:**
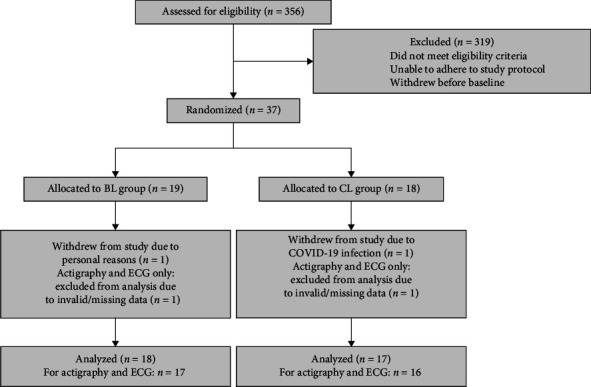
Participant flow diagram.

The final sample comprised 35 students (29 female, 6 male), with 18 (4 male) assigned to the BL group and 17 (2 male) to the CL group. Block randomization was done with the GraphPad online randomization tool (https://www.graphpad.com/quickcalcs/randomize1/). Due to the COVID-19 pandemic and corresponding lockdowns, we were not able to reach our planned sample size, as many participants canceled at short notice due to a COVID-19 infection. Additionally, due to quarantine regulations, across extended periods of time, only one participant was allowed to stay in the laboratory, and during total lockdowns, we were not able to collect any data. We, therefore, consider this study as a pilot study.  Figure 3 shows the participant flow diagram, and the completed CONSORT checklist is available in the Supporting Information 3 [116].

The mean age of the students was 22.4 ± 2.6 years, ranging from 18 to 29 years. Most participants (26) were medical students, while the remainder were studying pharmacy, physics, and law. Descriptive statistics from the screening questionnaire and the sample characterization questionnaire are presented in Table 1. No significant differences were observed between the groups for these parameters.

### 2.6. Statistics

Data are presented as mean ± standard deviation, with the minimum and maximum in square brackets. Nonnormally distributed data are presented as median, with the lower and upper quartile in parentheses and the minimum and maximum in square brackets. Figures display the mean with 95% confidence intervals unless specified otherwise.

We conducted two-way mixed analyses of variance (ANOVA) with the light intervention group as between-subjects factor and measurement time as the repeated measures factor. For the cognitive performance tasks and HRV parameters during these tasks, three-way mixed ANOVAs with two repeated measures factors (study day and measurement time per study day) were used. When sphericity was violated, the Greenhouse-Geisser correction was applied. Post hoc pairwise comparisons were performed using the Bonferroni–Holm correction to adjust for multiple comparisons. Data that violated parametric test assumptions were additionally analyzed using nonparametric alternatives, such as the Friedman test, Mann–Whitney *U* test, and Wilcoxon test. If nonparametric analyses confirmed the ANOVA results, only the ANOVA results are reported. The Bonferroni correction was also applied in nonparametric analyses to adjust for multiple comparisons.

Outliers were defined as absolute scores larger than 1.5 times the interquartile range (IQR). Outliers and missing data (resulting from user error or technical issues) were substituted with the mean value of the respective light intervention group. Overall, 5.44% of data were substituted.

All statistical tests were two-sided, with the significance level set at *α* = 0.05. Analyses were performed using JASP version 0.19.1 [117].

## 3. Results

### 3.1. Primary Outcome Parameters

Descriptive statistics for all primary outcome parameters are presented in Table 2.

#### 3.1.1. Test Anxiety (PAF-S)

**Figure 4:**
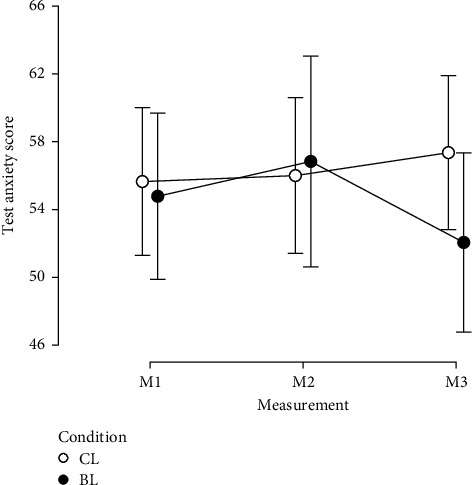
Test anxiety score. BL, bright light; CL, control light; M1, measurement 1 (Monday morning); M2, measurement 2 (Wednesday morning); M3, measurement 3 (Saturday morning).

A significant interaction effect between measurement time and light intervention group was found in the standardized PAF-S scores (F2, 66) = 4.99, *p*=0.010, *η*_p_^2^ = 0.131). Test anxiety scores significantly decreased from M2 to M3 under BL (*p*=0.001, *d* = 0.478), while they remained unchanged under CL (*p*=0.115, *d* = −0.135; Figure 4). However, the difference between the two light intervention groups at M3 was moderate but not significant (*p*=0.119, *d* = 0.530). Slight increases in test anxiety from M1 to M2 were observed in both groups; however, these were not significant (CL: *p*=0.827, *d* = 0.035; BL: *p*=0.195, *d* = −0.206). The main effects of measurement time (*F* (2, 66) = 1.54, *p*=0.222, *η*_p_^2^ = 0.045) and group (*F* (1, 33) = 0.31, *p*=0.580, *η*_p_^2^ = 0.009) were not significant.

#### 3.1.2. Anxiety (STAI-S)

We found a significant main effect of group (*F* (1, 33) = 22.06, *p* < 0.001, *η*_p_^2^ = 0.401), with consistently higher scores in the CL group. However, there was no significant main effect of measurement (*F* (2, 66) = 0.61, *p*=0.547, *η*_p_^2^ = 0.018) or interaction effect (*F* (2, 66) = 2.13, *p*=0.127, *η*_p_^2^ = 0.061).

#### 3.1.3. Psychological Distress (BSI-18)

No significant main effects were found for group (*F* (1, 33) = 2.78, *p*=0.105, *η*_p_^2^ = 0.078) or measurement (*F* (2, 66) = 0.34, *p*=0.711, *η*_p_^2^ = 0.010) and no significant interaction effect (*F* (2, 66) = 1.21, *p*=0.305, *η*_p_^2^ = 0.035) regarding the BSI-18 score.

### 3.2. Secondary Outcome Parameters

#### 3.2.1. Working Memory (2-Back)

Descriptive statistics of 2-back data are presented in Supporting Information 4: Table S2.

The 2-back scores showed no significant differences between the two light intervention groups at any measurement time (T1: *U* = 171.00, *p*=0.563, *r* = 0.118; T2: *U* = 112.00, *p*=0.179, *r* = −0.268; T3: *U* = 168.00, *p*=0.631, *r* = 0.098; T4: *U* = 176.50, *p*=0.447, *r* = 0.154; T5: *U* = 149.00, *p*=0.908, *r* = −0.026; T6: *U* = 164.00, *p*=0.728, *r* = 0.072; T7: *U* = 120.50, *p*=0.290, *r* = −0.212; T8: *U* = 188.00, *p*=0.252, *r* = 0.229; T9: *U* = 125.50, *p*=0.371, *r* = −0.180). However, Friedman tests indicated significant differences between measurement points in both groups (CL: *χ*^2^ = 41.92, *p* < 0.001, *W* = 0.308; BL: *χ*^2^ = 57.13, *p* < 0.001, *W* = 0.397).

In the CL group, there were no significant changes in the 2-back score during the first six measurements (T1-T2: *z* = 1.03, *p*=0.313, *r* = 0.294; T1-T3: *z* = −1.58, *p*=0.121, *r* = −0.449; T4-T5: *z* = 0.75, *p*=0.468, *r* = 0.213; T4-T6: *z* = −1.88, *p*=0.065, *r* = −0.615). On the fifth study day, scores increased significantly from T7 to T8 (*z* = −2.81, *p*=0.005, *r* = −0.825), but not from T7 to T9 (*z* = −1.68, *p*=0.099, *r* = −0.492). Interestingly, comparisons between the initial measurement on day 1 (T1) and the final measurement on day 5 (T9) revealed improved 2-back scores (T1-T9: *z* = −2.20, *p*=0.029, *r* = −0.608). However, other key time points in the CL group showed no significant changes (T1-T7: *z* = −1.42, *p*=0.163, *r* = −0.404; T3-T9: *z* = −1.56, *p*=0.125, *r* = −0.458).

In the BL group, scores did not change on the first day (T1-T2: *z* = −0.98, *p*=0.338, *r* = −0.279; T1-T3: *z* = −1.48, *p*=0.144, *r* = −0.398). On the second day, scores increased significantly from T4 to T6 (*z* = −2.53, *p*=0.012, *r* = −0.699), but not from T4 to T5 (*z* = −0.43, *p*=0.691, *r* = −0.125). On the third day, scores increased significantly from T7 to T9 (*z* = −2.07, *p*=0.040, *r* = −0.608), but not from T7 to T8 (*z* = −0.69, *p*=0.507, *r* = −0.190). Direct comparisons between the first day and fifth day revealed significant increases in scores from T1 to T9 (*z* = −3.57, *p* < 0.001, *r* = −0.987), T1 to T7 (*z* = −3.10, *p*=0.002, *r* = −0.856), and T3 to T9 (*z* = −3.52, *p* < 0.001, *r* = −1.000).

#### 3.2.2. Response Inhibition (GNT)

Descriptive statistics of GNT data are presented in Supporting Information 4: Table S3.

Regarding GNT reaction speed, we found a significant main effect of the day (*F* (2, 66) = 4.78, *p*=0.012, *η*_p_^2^ = 0.126), with significantly higher reaction speeds on the third day compared to the first day (*p*=0.006, *d* = −0.274), regardless of measurement time or light intervention group. The main effects of measurement time (*F* (2, 66) = 0.002, *p*=0.998, *η*_p_^2^ = 0.000) and light intervention group (*F* (1, 33) = 1.26, *p*=0.270, *η*_p_^2^ = 0.037) were not significant. There was a significant interaction effect for day x measurement x group (*F* (4, 132) = 3.33, *p*=0.012, *η*_p_^2^ = 0.092), but no significant interaction effects of day x group (*F* (2, 66) = 1.03, *p*=0.363, *η*_p_^2^ = 0.030), measurement x group (*F* (2, 66) = 0.38, *p*=0.685, *η*_p_^2^ = 0.011), or day x measurement (*F* (4, 132) = 0.43, *p*=0.786, *η*_p_^2^ = 0.0013). Post-hoc tests indicated that only three out of 162 pairwise comparisons reached significance.

For the GNT commission error rate, no significant differences over time were observed in either the CL group (*χ*^2^ = 11.98, *p*=0.152, *W* = 0.088) or the BL group (*χ*^2^ = 5.07, *p*=0.750, *W* = 0.035).

#### 3.2.3. Presleep Arousal and Self-Reported Sleep Quality

Descriptive statistics of presleep arousal and self-reported sleep quality are presented in Table S4.

Regarding presleep arousal, no significant differences were found between groups at any measurement point (N1: *U* = 158.00, *p*=0.882, *r* = 0.033; N2: *U* = 146.50, *p*=0.843, *r* = −0.042; N3: *U* = 141.50, *p*=0.715, *r* = −0.075; N4: *U* = 101.50, *p*=0.092, *r* = −0.337; N5: *U* = 209.50, *p*=0.064, *r* = 0.369). Within-group analysis using Friedman tests showed significant changes in the CL group (*χ*^2^ = 14.04, *p*=0.007, *W* = 0.206), but not in the BL group (*χ*^2^ = 8.38, *p*=0.079, *W* = 0.116). Pairwise comparisons in the CL group (using Bonferroni-correction) revealed no significant results, though there was a marginally not-significant decrease in presleep arousal from N1 to N2 (*z* = 2.69, *p*=0.007, *r* = 0.765) and a marginally not-significant increase from N4 to N5 (*z* = −2.77, *p*=0.006, *r* = −0.787).

For self-reported sleep quality, no significant differences were found between groups (N1: *U* = 181.50, *p*=0.327, *r* = 0.186; N2: *U* = 155.50, *p*=0.944, *r* = 0.016; N3: *U* = 187.50, *p*=0.230, *r* = 0.225; N4: *U* = 165.00, *p*=0.688, *r* = 0.078; N5: *U* = 147.00, *p*=0.850, *r* = −0.039). Within-group Friedman tests also yielded no significant results (CL: *χ*^2^ = 7.73, *p*=0.102, *W* = 0.114; BL: *χ*^2^ = 7.03, *p*=0.134, *W* = 0.098).

#### 3.2.4. Objective Sleep Quality

Descriptive statistics of actigraphy data are presented in Table S5. Due to technical errors, two actimeters (one from each light intervention group) failed to record any data, reducing the total sample for actigraphy analysis to 33.

Analysis of sleep efficiency data revealed an interaction effect between night and light intervention group that was nearly significant (*F* (4, 124) = 2.44, *p*=0.050, *η*_p_^2^ = 0.073). There were no significant main effects for night (*F* (4, 124) = 1.12, *p*=0.348, *η*_p_^2^ = 0.035) or light intervention (*F* (1, 31) = 0.19, *p*=0.665, *η*_p_^2^ = 0.006).

Analysis of total sleep time showed no significant interaction effect (*F* (4, 124) = 1.68, *p*=0.160, *η*_p_^2^ = 0.051), nor were there significant main effects for condition (*F* (1, 31) = 0.27, *p*=0.609, *η*_p_^2^ = 0.009) or night (*F* (4, 124) = 2.14, *p*=0.080, *η*_p_^2^ = 0.064).

**Figure 5:**
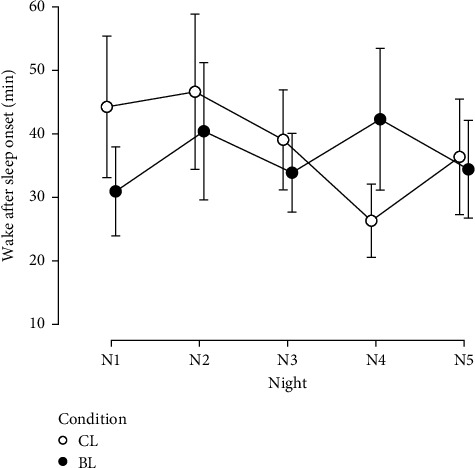
Wake after sleep onset. BL, bright light; CL, control light; N1–5, night 1–5.

For wake time after sleep onset (WASO), a significant interaction effect was found (*F* (4, 124) = 4.97, *p* < 0.001, *η*_p_^2^ = 0.138). Post hoc test revealed significant differences between groups on night one (N1: *p*=0.037, *d* = 0.762) and night four (N4: *p*=0.013, *d* = −0.915), with lower WASO in the BL group on N1 but higher WASO on N4 (Figure 5). No significant differences between the two groups were observed on nights 2, 3, and 5. In the CL group, WASO on N4 was significantly lower than on N1 (*p*=0.026, *d* = 1.027), N2 (*p*=0.009, *d* = 1.163), and N3 (*p*=0.050, *d* = 0.730). In addition, no significant main effect was found for night (*F* (4, 124) = 2.15, *p*=0.078, *η*_p_^2^ = 0.065) and condition (*F* (1, 31) = 0.26, *p*=0.616, *η*_p_^2^ = 0.008).

Regarding the number of awakenings, no significant interaction effect was found (*F* (4, 124) = 0.40, *p*=0.806, *η*_p_^2^ = 0.013), nor were there significant main effects for night (*F* (4, 124) = 1.12, *p*=0.350, *η*_p_^2^ = 0.035) or condition (*F* (1, 31) = 0.78, *p*=0.383, *η*_p_^2^ = 0.025).

**Figure 6:**
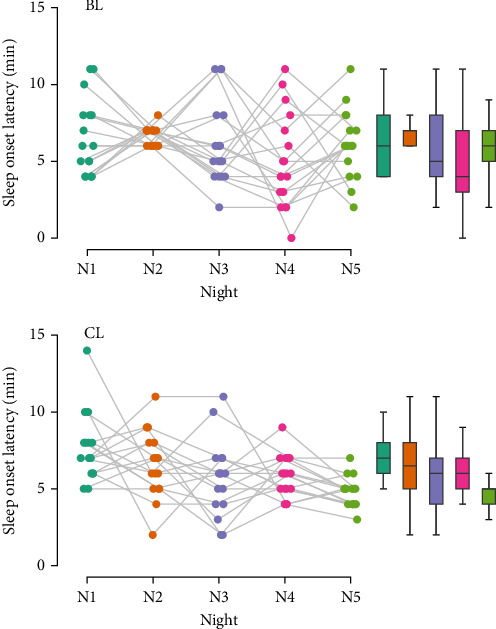
Raincloud plot of sleep onset latency. BL, bright light; CL, control light; N1–5, night 1–5.

Finally, no significant differences between groups were observed in sleep onset latency (using Bonferroni-correction) at any measurement point (N1: *U* = 178.00, *p*=0.130, *r* = 0.309; N2: *U* = 126.00, *p*=0.721, *r* = −0.074; N3: *U* = 129.50, *p*=0.827, *r* = −0.048; N4: *U* = 173.00, *p*=0.184, *r* = 0.272; N5: *U* = 76.00, *p*=0.029, *r* = −0.441). However, sleep onset latency decreased significantly throughout the study in the CL group only (CL: *χ*^2^ = 23.53, *p* < 0.001, *W* = 0.368; BL: *χ*^2^ = 7.72, *p*=0.102, *W* = 0.114). Pairwise comparisons in the CL group with a Bonferroni-adjusted alpha revealed a significant decrease from N1 to N5 (*z* = 3.41, *p* < 0.001, *r* = 1.000). However, comparisons from N1 to N2 (*z* = 1.05, *p*=0.303, *r* = 0.308), N2 to N3 (*z* = 1.26, *p*=0.219, *r* = 0.396), N3 to N4 (*z* = −0.38, *p*=0.727, *r* = −0.114), and N4 to N5 (*z* = 2.47, *p*=0.011, *r* = 0.725) did not show significant results (Figure 6).

Descriptive statistics of HRV parameters during sleep are presented in Table S8, with ANOVA results summarized in Table 3. Due to low data quality, two participants (one from each light intervention group) were excluded from these analyses, resulting in a sample size of 33. No significant results were found in these analyses.

#### 3.2.5. Physiological Stress Markers During Cognitive Performance Tasks

All details, including descriptive statistics and additional analyses of HRV parameters during the cognitive tasks, are provided in the Supporting Information 4: Tables S6–S10. To summarize, we observed a significant main effect of the light intervention group during both cognitive performance tasks, with a lower average heart rate in the BL group. Heart rate and LF/HF ratio improved significantly (i.e., indicated lower stress levels) in both groups over the course of each laboratory visit. During the GNT, a significant interaction between day and measurement time was found for these parameters.

Nonparametric analyses revealed significant effects of measurement time on SDNN and RMSSD. For RMSSD, Mann–Whitney *U* tests additionally indicated significant group differences during both cognitive tasks on the first day (T2 and T3), with higher values in the BL group (indicating lower stress) but not on the fifth day (T8 and T9).

Within-group analyses showed that RMSSD increased significantly in the BL group during the GNT from T1 to T2 and from T1 to T3 but not between T2 and T3. In contrast, the CL group showed no significant changes across these intervals. On the fifth day, both groups showed significant increases in RMSSD scores from T7 to T8 and from T7 to T9, but not from T8 to T9.

During the 2-back task, RMSSD increased significantly in both groups, from T1 to T2, and in the BL group, also from T1 to T3. No significant changes were observed from T2 to T3 in either group. As with the GNT, both groups demonstrated significant increases in RMSSD scores from T7 to T8 and from T7 to T9, but not from T8 to T9.

### 3.3. Tertiary Outcome Parameters

Descriptive statistics of tertiary outcome parameters are presented in Table 4.

#### 3.3.1. Perceived Usefulness of the Workplace Luminaire

Mann–Whitney *U* tests revealed no significant difference between groups regarding the expected usefulness of the workplace luminaire before study participation (*U* = 121.00, *p*=0.249, *r* = −0.209). However, after participating in the study, the BL group was significantly more inclined to use the workplace luminaire again (*U* = 63.50, *p*=0.002, *r* = −0.585), recommend its usage to a friend in a similar exam preparation period (*U* = 56.00, *p* < 0.001, *r* = −0.634), and felt significantly better supported while learning for the exam (*U* = 70.00, *p*=0.004, *r* = −0.542).

#### 3.3.2. Academic Performance

No significant differences were found between groups regarding academic performance, as measured by the difference between the expected and actual grades (*U* = 129.50, *p*=0.423, *r* = 0.154).

## 4. Discussion

This study examined the effects of a 5-day evening BL intervention on test anxiety, stress, cognitive function, sleep quality, and HRV in university students during exam preparation. The results partially supported our hypotheses.

### 4.1. Test Anxiety, General Anxiety, and Psychological Distress

This study found a moderate significant reduction in test anxiety in the BL group from the second to the third measurement, indicating a potential benefit of BL exposure for test anxiety. It should be noted that both groups showed a small but nonsignificant increase in test anxiety from M1 to M2, with a slightly higher increase in the BL group. We expected that test anxiety might increase during the study [118], and it is also possible that the effects of the light intervention may only become apparent with a delay. Further research is needed to investigate the possibility of delayed nonvisual light effects.

Although the STAI-S results showed no significant interaction effect, anxiety levels were consistently higher in the CL group, complicating interpretation. STAI-T data, collected a week before the study, showed no significant difference between groups, suggesting that while the CL group experienced more acute state anxiety during the study, their trait anxiety was similar to that of the BL group. Previous research on BL's effect on general anxiety is mixed: Some studies report reduced anxiety [51, 53, 112], while others find no effect [46, 113], especially in high-anxiety individuals [52].

The BSI-18 results showed no significant effects of BL exposure on psychological distress. To our knowledge, no previous studies have used this measure to assess light exposure effects.

### 4.2. Cognitive Performance

The 2-back task results showed significant improvements in working memory performance over time, independent of the light condition, indicating a practice effect rather than a specific impact of BL exposure. This practice effect was expected, as documented in the literature [93, 110]. Although participants practiced the test before the study, this did not fully eliminate the practice effect. Nevertheless, the lack of significant differences between groups suggests that BL did not provide additional benefits for working memory. Evidence on BL's effect on working memory is mixed, with some studies finding no effects [96, 97], some adverse effects [95], and others beneficial effects [98, 99]. Variability in how BL is applied across studies, such as differences in light intensity, CCT, exposure duration, and timing, as well as general differences in study designs, may explain conflicting findings [56, 57]. While recent guidelines suggest standardized reporting of light exposure, they are not yet widely followed [119–121]. Additionally, small sample sizes and varied measurement methods complicate comparisons between studies.

For the GNT, no significant differences were found in reaction speed or commission error rate between groups or over time, consistent with other studies showing no significant effects of BL [122, 123].

### 4.3. Presleep Arousal and Sleep Quality

Contrary to concerns about evening BL disrupting sleep [62, 63, 124], we found no significant differences between groups in presleep arousal, self-reported sleep quality, or most actigraphically measured sleep parameters. This supports our hypothesis and suggests that the timing and duration of our intervention (ending at 10:30 p.m.) were appropriate to avoid negative sleep impacts, aligning with studies suggesting earlier evening light exposure mitigates these effects [64].

Actigraphy data revealed a significant decrease in sleep onset latency in the CL group from the first to the fifth night, indicating a mild sleep-promoting effect of dimmer evening light. However, no significant differences were found between groups, and importantly, the intervention group did not experience increased sleep onset latency as might be expected based on the literature. These results are complemented by HRV analyses during sleep, supporting the notion that bright evening light had no negative impact on nighttime sleep. Further research is needed to understand this finding.

### 4.4. Physiological Stress Markers During Cognitive Performance Tasks

During both cognitive tasks (2-back and go/no-go), the BL group had significantly lower heart rates than the CL group. The difference is present at T1 before the light intervention began and is likely due to inherent group differences. We observed significant improvements in all HRV parameters over time, independent of the experimental group, indicating increased parasympathetic activity (lower stress) from the first to the second measurement within each session, with some parameters improving further by the third measurement. This pattern was consistent on both the first and fifth days, suggesting a general relaxation effect as participants became more comfortable with the tasks. This result is in line with previous findings showing increased parasympathetic activity during repeated cognitive tasks [97, 125].

Analysis of RMSSD, reflecting short-term HRV [109] showed significant differences between groups during cognitive tasks on the first day but not on the fifth day. The BL group exhibited significant RMSSD increases from the first to second and third measurements on the first day, while the CL group did not, suggesting that BL exposure may have initially facilitated a quicker relaxation response during cognitive tasks, though this effect was not sustained throughout the study.

### 4.5. Subjective Ratings and Academic Performance

Participants in the BL group perceived the workplace luminaire as significantly more useful than those in the CL group after the study. They were also more likely to report that they would use the luminaire again and recommend it to others during high-stress exam periods. This result suggests that students subjectively benefited from BL exposure, even if not all objective measures showed significant improvements.

However, no significant differences were found between groups in academic performance, as measured by the difference between expected and actual grades. While BL exposure may have positive psychological effects, it may not directly enhance exam performance.

### 4.6. Limitations and Future Directions

Several limitations of this study should be noted. First, the relatively small sample size may have limited our ability to detect smaller effects. Conducting part of the study during the COVID-19 pandemic posed challenges, as restrictions sometimes allowed only one participant in the lab at a time. Consequently, we could not include the number of subjects outlined in the preregistered protocol. Due to technical problems, ECG and actigraphy data could not be recorded for one participant in each group. These individuals were excluded from analyses, which further reduced our sample size. Additionally, our sample was predominantly female and mostly comprised medical students, which limits the generalizability of the results. Future studies with larger and more diverse samples could provide more definitive conclusions about the efficacy of evening BL interventions for students. Second, the 1-week study duration may not have been sufficient to capture all potential light effects. Circadian disruption is one of the main concerns with respect to late-night BL exposure. Long-term studies could monitor possible circadian shifts with direct biological measures (e.g., dim light melatonin onset) or indirect indicators (e.g., changes in mid-sleep time or daytime fatigue). Additionally, it could be explored whether the effects of BL exposure accumulate over time or if there are delayed effects, including on sleep. Third, confounding factors such as prestudy sleep patterns should be assessed, as short-term changes might be undetectable otherwise; we only monitored sleep starting after the first light exposure. Participant chronotype may moderate the effects of an evening BL exposure. This possibility should be explored in future studies. Fourth, personal light exposure outside the lab should be monitored. Although we used wrist-worn actimeters while also recorded light levels, from the first evening, the light sensors were often covered by clothing during winter, reducing data quality. Fifth, baseline differences in anxiety levels (STAI-S) between groups might mask the potential effects of BL exposure. Future studies should pay special attention to stratifying groups during the screening and group allocation process with regard to perceived test anxiety and psychological distress. Similarly, practice effects in the 2-back task could obscure possible light effects. Sixth, we only assessed the perceived usefulness of the lighting intervention at the end of the study. Additionally, assessing it immediately after the first study session would enable researchers to capture changes in perceived usefulness over the course of the study.

## 6. Conclusions

In conclusion, our findings suggest that an evening BL intervention may be beneficial for students during intensive learning periods. The study indicates mild anxiolytic effects after several days of evening light exposure, though no immediate cognitive effects were observed. Importantly, these effects were achieved without disrupting sleep patterns, as evidenced by both subjective (self-report questionnaires) and objective (actigraphy, HRV) measures.

The perceived usefulness of the BL intervention suggests that it could be a well-accepted supportive measure for students during stressful exam periods. However, the lack of significant effects on other measures, such as psychological distress and cognitive performance, underscores the need for further research to fully understand the potential of BL interventions as a countermeasure for stress and anxiety in university populations.

## Figures and Tables

**Table 1:** Descriptive statistics of the screening and characterization questionnaire.

Questionnaire	BL	CL	*p*-Value
Screening questionnaires			
MSF_sc_	04:18 ± 01:09 [02:08–07:02]	04:18 ± 01:02 [02:13–06:00]	0.996
TAI-G	76.50 ± 14.08 [60–107]	76.00 ± 10.46 [60–91]	0.906
PHQ-9	7.11 ± 2.93 [4–14]	7.29 ± 4.10 [1–14]	0.880
Characterization questionnaires			
ADS-L	17.00 ± 6.84 [6–33]	20.88 ± 7.11 [12–39]	0.121
PIDS-SA Part 1	3.82 ± 2.10 [1–8]	4.19 ± 2.46 [0–7]	0.650
PIDS-SA Part 2	7.12 ± 3.72 [0–12]	5.69 ± 3.59 [1–13]	0.271
PIDS-SA Part 4	4.12 ± 2.64 [0–8]	3.88 ± 1.96 [1–8]	0.768
STAI-T	43.19 ± 11.26 [27.14–70.00]	48.93 ± 10.99 [31.43–72.86]	0.149
PSQI	5.71 ± 2.23 [2–11]	5.88 ± 2.58 [1–9]	0.841
PSS-10	26.94 ± 6.42 [15–38]	30.00 ± 6.14 [21–40]	0.172
PSS-10-H	16.41 ± 4.26 [9–23]	18.13 ± 3.54 [13–23]	0.220
PSS-10-S	13.47 ± 2.83 [9–18]	12.13 ± 3.28 [7–18]	0.216

*Note*: Data show the mean ± standard deviation with minimum and maximum in square brackets. *T*-tests were conducted to check for differences between groups. ADS-L, General Depression Scale; MSF_sc_, midpoint of sleep on free days (corrected for sleep debt); PHQ-9, Patient Health Questionnaire; PIDS-SA, Personal Inventory for Depression and SAD; PSS-10, Perceived Stress Scale-total score; PSS-10-H, Perceived Stress Scale-helplessness; PSS-10-S, Perceived Stress Scale-lack of self-efficacy; STAI-T, State–Trait-Anxiety Inventory-Trait; TAI-G, Test Anxiety Questionnaire.

Abbreviations: BL, bright light; CL, control light; PSQI, Pittsburgh Sleep Quality Index.

**Table 2:** Descriptive statistics for primary outcome parameters.

Measurement	PAF-S	
	BL	CL
M1	54.78 ± 9.85 [37–72]	55.65 ± 8.48 [39–71]
M2	58.83 ± 12.49 [37–83]	56 ± 8.93 [39–81]
M3	52.06 ± 10.63 [30–73]	57.35 ± 8.82 [40–75]

	**STAI-S**	
	**BL**	**CL**

M1	42.56 ± 6.92 [31–54]	46.94 ± 3.40 [40–55]
M2	43.06 ± 6.83 [32–59]	48.06 ± 5.84 [40–62]
M3	40.17 ± 2.66 [33–44]	48.65 ± 4.00 [43–57]

	**BSI-18**	
	**BL**	**CL**

M1	7.67 ± 4.47 [0–15]	8.00 ± 2.18 [4–14]
M2	7.11 ± 4.07 [1–15]	9.94 ± 5.89 [2–23]
M3	7.11 ± 4.48 [0–16]	9.35 ± 3.77 [3–17]

*Note*: Data show the mean ± standard deviation with minimum and maximum in square brackets. BSI-18, Brief Symptom Inventory; M1, measurement 1 (Monday morning); M2, measurement 2 (Wednesday morning); M3, measurement 3 (Saturday morning); PAF-S, Test Anxiety Inventory; STAI-S, State–Trait Anxiety Inventory-State.

Abbreviations: BL, bright light; CL, control light.

**Table 3:** Summary of ANOVA results (*p*-values) of HRV parameters during sleep.

Parameter	Restful sleep			Restless sleep		
	Group	Night	Night × Group	Group	Night	Night × Group
HR	0.260	0.933	0.894	0.208	0.570	0.450
SDNN	0.553	0.104	0.182	0.932	0.564	0.265
RMSSD	0.595	0.441	0.092	0.718	0.644	0.097
LF/HF	0.932	0.063	0.277	0.514	0.484	0.156

*Note:* LF/HF, ratio between LF and HF; RMSSD, square root of the mean squared differences of successive R–R intervals; SDNN, standard deviation of R–R intervals.

Abbreviations: HF, high frequency; HR, heart rate; LF, low frequency.

**Table 4:** Descriptive statistics of tertiary outcome parameters.

Parameter	BL	CL
Expected usefulness	4 (4–4) [3–5]	4 (3–4) [2–5]
Use again	5 (4–5) [2–5]	3 (3–4) [2–5]
Recommend	5 (5–5) [2–5]	3 (3–4) [2–5]
Helpfulness	5 (4–5) [2–5]	3 (3–4) [2–5]
Grade diff	0 (−1 to 1) [−2 to 2]	0 (0–1) [−2 to 2]

*Note*: Data show the median with lower and upper quartile in parentheses and minimum and maximum in square brackets. Actual Usefulness, “I think that the study workplace with the luminaire helped me to study during an intensive exam preparation period.” These questions were answered on a scale of 1–5; Expected Usefulness, “I think that the study workplace with the luminaire can help me to study during an intensive exam preparation period.”; Grade Diff, difference between expected grade and received grade. Positive values denote better grades than expected, negative values worse grades; Recommend, “I would recommend the study workplace with the luminaire to friends who are in an intensive exam preparation period.”; Use Again, “I would use the study workplace with the luminaire again when I am in an intensive exam preparation period.”

Abbreviations: BL, bright light; CL, control light.

## Data Availability

The data that support the findings of this study are available from the corresponding author upon reasonable request.

## Supporting Information

Supporting Information 1 Spectrum_CL: Spectral measurement data for each workplace during the CL condition.

Supporting Information 2 Spectrum_BL: Spectral measurement data for each workplace during the BL condition.

Supporting Information 3 CONSORT_Checklist: CONSORT checklist for this study.

Supporting Information 4 In the .docx file “ReducingExamAnxiety_SupportingInformation,” Tables S1–S11, Figures S1 and S2, and the results regarding physiological stress markers during cognitive performance tasks are available.
