# Supplementary material for: Reducing Test Anxiety: A Randomized Controlled Pilot Study of Evening Bright Light Exposure in University Students
Source: Depress Anxiety. 2025 Aug 26;2025:1422406. doi: 10.1155/da/1422406 (PMC12404836; doi:10.1155/da/1422406)

**Supporting Information**

**Reducing Test Anxiety: A Randomized Controlled Trial of Evening Bright Light Exposure in University Students**

Maximilian Dick^1,2^*, Helmut K. Lackner^3^, Elisabeth M. Weiss^1^, Markus Canazei^1^

^1^ Department of Psychology, University of Innsbruck, Innsbruck, Austria

^2^ Research and Development Department, Bartenbach GmbH, Aldrans, Austria

^3^ Division of Physiology, Otto Loewi Research Center, Medical University of Graz, Graz, Austria

* Corresponding author

E-mail: [Maximilian.Dick@bartenbach.com](mailto:Maximilian.Dick@bartenbach.com) (MD)

# S1 Overview and timing of the questionnaires

Table S1. Overview and timing of the questionnaires

| **Questionnaire** | **Measures** | **Timing** |
| --- | --- | --- |
| Screening | TAI-G, μMCTQ, PHQ-9, BDI-II | Up to 1 month before participation |
| Characterization | ADS-L, PIDS-SA, STAI-T, PSQI, PSS-10 | 1 week before participation |
| Outcome | PAF-S, BSI-18, STAI-S | First, third, and sixth morning of the study |
| Sleep | PSAS, Sleep quality, bedtimes | Every morning during the study |
| Follow-Up | Exam grade | 2-3 weeks after the study |

TAI-G = Test Anxiety Inventory; μMCTQ = Munich ChronoType Questionnaire; PHQ-9 = Patient Health Questionnaire; BDI-II = Beck Depression Inventory II; ADS-L = General Depression Scale; PIDS-SA = Personal Inventory for Depression and SAD; STAI-T = State-Trait Anxiety Inventory – Trait; PSQI = Pittsburgh Sleep Quality Index; PSS-10 = Perceived Stress Scale; PAF-S = Test Anxiety Questionnaire; BSI-18 = Brief Symptom Inventory; STAI-S = State-Trait Anxiety Inventory – State; PSAS = Pre-Sleep Arousal Scale

# S2 Cognitive performance tasks

Table S2. Descriptive statistics of 2-back data

|  | **2-back score** | |
| --- | --- | --- |
|  | **BL** | **CL** |
| **T1** | 4.17 (3.50-4.79) [2.00-5.50] | 4.33 (4.00-5.00) [2.00-5.67] |
| **T2** | 4.33 (4.00-4.67) [3.33-5.50] | 4.17 (3.33-4.67) [2.50-5.17] |
| **T3** | 4.42 (3.54-5.17) [1.83-5.83] | 4.50 (4.99-5.17) [3.50-6.00] |
| **T4** | 4.59 (3.92-5.13) [2.67-6.00] | 4.83 (4.50-5.17) [3.17-5.83] |
| **T5** | 4.57 (4.04-5.50) [2.67-5.83] | 4.67 (3.83-5.50) [2.83-6.00] |
| **T6** | 4.88 (4.71-5.29) [3.83-5.83] | 5.00 (4.67-5.33) [3.33-6.00] |
| **T7** | 5.00 (4.58-5.46) [3.17-6.00] | 4.67 (4.00-5.33) [2.33-6.00] |
| **T8** | 5.00 (4.58-5.50) [3.83-6.00] | 5.17 (5.17-5.50) [4.33-6.00] |
| **T9** | 5.28 (5.00-5.63) [3.83-6.00] | 4.83 (4.17-5.50) [2.83-6.00] |

Data show the median with lower and upper quartile in parentheses and minimum and maximum in square brackets; BL = bright light; CL = common lighting; T1-9 = measurement time 1-9: T1-T3 were on the first day, T4-T6 on the third day, T7-T9 on the fifth day. T1, T4 and T7 were immediately before the onset of the light intervention, T2, T5, and T8 were during the light intervention, T3, T6 and T9 were immediately after the end of the light intervention.

Table S3. Descriptive statistics of GNT data

|  | **GNT speed** | |
| --- | --- | --- |
|  | **BL** | **CL** |
| **T1** | 3.32 ± 0.38 [2.74-4.06] | 3.34 ± 0.45 [2.59-4.15] |
| **T2** | 3.34 ± 0.42 [2.59-4.07] | 3.27 ± 0.31 [2.73-3.77] |
| **T3** | 3.39 ± 0.35 [2.90-3.89] | 3.26 ± 0.49 [2.53-4.36] |
| **T4** | 3.44 ± 0.41 [2.66-4.24] | 3.38 ± 0.41 [2.68-4.18] |
| **T5** | 3.57 ± 0.24 [3.14-4.12] | 3.32 ± 0.22 [3.06-3.92] |
| **T6** | 3.48 ± 0.34 [2.85-4.20] | 3.35 ± 0.26 [2.80-4.01] |
| **T7** | 3.50 ± 0.36 [2-94-4.23] | 3.29 ± 0.50 [2.46-4.29] |
| **T8** | 3.41 ± 0.25 [2.99-3.84] | 3.36 ± 0.33 [2.59-4.00] |
| **T9** | 3.49 ± 0.31 [2.88-3.95] | 3.31 ± 0.49 [2.48-4.39] |
|  | **GNT commission error rate** | |
|  | **BL** | **CL** |
| **T1** | 0.00 (0.00-0.02) [0.00-0.03] | 0.00 (0.00-0.02) [0.00-0.05] |
| **T2** | 0.00 (0.00-0.02) [0.00-0.05] | 0.00 (0.00-0.02) [0.00-0.07] |
| **T3** | 0.02 (0.00-0.02) [0.00-0.05] | 0.00 (0.00-0.02) [0.00-0.08] |
| **T4** | 0.00 (0.00-0.02) [0.00-0.05] | 0.01 (0.00-0.02) [0.00-0.03] |
| **T5** | 0.01 (0.00-0.03) [0.00-0.03] | 0.02 (0.00-0.02) [0.00-0.07] |
| **T6** | 0.02 (0.00-0.02) [0.00-0.03] | 0.02 (0.00-0.02) [0.00-0.08] |
| **T7** | 0.00 (0.00-0.02) [0.00-0.05] | 0.00 (0.00-0.02) [0.00-0.08] |
| **T8** | 0.00 (0.00-0.02) [0.00-0.03] | 0.02 (0.00-0.02) [0.00-0.05] |
| **T9** | 0.00 (0.00-0.01) [0.00-0.03] | 0.03 (0.00-0.03) [0.00-0.11] |

Reaction speed data show the mean ± standard deviation with minimum and maximum in square brackets; error rate data show the median with lower and upper quartile in parentheses and minimum and maximum in square brackets; BL = bright light; CL = common lighting; GNT = go-/no-go task; T1-9 = measurement time 1-9: T1-T3 were on the first day, T4-T6 on the third day, T7-T9 on the fifth day. T1, T4 and T7 were immediately before the onset of the light intervention, T2, T5, and T8 were during the light intervention, T3, T6 and T9 were immediately after the end of the light intervention.

# S3 Sleep-related variables

Table S4. Descriptive statistics of pre-sleep arousal and self-reported sleep quality

|  | **PSAS** | |
| --- | --- | --- |
|  | **BL** | **CL** |
| **N1** | 26.50 (21.25-34.00) [17.00-47.00] | 28.00 (24.00-31.00) [21.00-37.00] |
| **N2** | 24.00 (19.50-31.00) [17.00-52.00] | 24.00 (21.00-27.00) [18.00-37.00] |
| **N3** | 25.00 (21.00-28.75) [15.00-32.00] | 24.00 (21.00-26.00) [16.00-43.00] |
| **N4** | 28.00 (23.50-33.00) [16.00-47.00] | 23.00 (19.00-29.00) [15.00-36.00] |
| **N5** | 25.00 (20.25-27.75) [15.00-36.00] | 29.00 (25.00-30.00) [18.00-45.00] |
|  | **Self-reported sleep quality** | |
|  | **BL** | **CL** |
| **N1** | 2.00 (2.00-3.00) [1.00-4.00] | 3.00 (2.00-3.00) [1.00-5.00] |
| **N2** | 2.00 (1.00-2.75) [1.00-4.00] | 2.00 (2.00-2.00) [1.00-3.00] |
| **N3** | 2.00 (1.00-2.00) [1.00-3.00] | 2.00 (2.00-3.00) [1.00-4.00] |
| **N4** | 2.00 (2.00-2.00) [1.00-5.00] | 3.00 (1.00-3.00) [1.00-4.00] |
| **N5** | 2.00 (2.00-3.00) [1.00-5.00] | 2.00 (1.00-3.00) [1.00-4.00] |

Data show the median with lower and upper quartile in parentheses and minimum and maximum in square brackets; PSAS = pre-sleep arousal scale; BL = bright light, CL = common lighting; N1-5 = night 1-5

Table S5. Descriptive statistics of actigraphy data

|  | **Sleep Efficiency [%]** | | **Total Sleep Time [min]** | |
| --- | --- | --- | --- | --- |
|  | **BL** | **CL** | **BL** | **CL** |
| **N1** | 91.46 ± 3.23 [85.87-96.16] | 88.64 ± 4.72 [79.79-95.71] | 404.00 ± 61.60 [309-496] | 421.13 ± 32.98 [374-494] |
| **N2** | 90.16 ± 4.26 [83.51-96.35] | 89.86 ± 3.43 [82.45-94.58] | 414.47 ± 58.66 [309-528] | 433.13 ± 56.28 [351-550] |
| **N3** | 90.46 ± 2.89 [84.87-94.51] | 89.51 ± 3.85 [81.14-94.12] | 399.47 ± 61.82 [303-518] | 423.25 ± 61.54 [310-548] |
| **N4** | 90.20 ± 4.27 [81.43-98.21] | 91.29 ± 3.49 [83.60-98.16] | 426.06 ± 51.58 [332-513] | 393.31 ± 67.61 [258-506] |
| **N5** | 90.71 ± 3.89 [83.71-97.61] | 91.64 ± 3.05 [85.83-96.61] | 435.41 ± 82.73 [300-571] | 445.69 ± 66.52 [339-570] |
|  | **Wake After Sleep Onset [min]** | | **Number of Awakenings** | |
|  | **BL** | **CL** | **BL** | **CL** |
| **N1** | 30.94 ± 13.65 [9-52] | 44.25 ± 20.89 [11-85] | 15.94 ± 7.29 [5-30] | 18.06 ± 6.04 [8-27] |
| **N2** | 40.41 ± 21.01 [11-70] | 46.63 ± 22.93 [16-101] | 18.24 ± 3.15 [11-25] | 20.56 ± 9.56 [9-37] |
| **N3** | 33.88 ± 12.06 [18-59] | 39.06 ± 14.75 [16-73] | 16.82 ± 4.76 [92-27] | 18.56 ± 6.72 [8-27] |
| **N4** | 42.29 ± 21.70 [5-89] | 26.31 ± 10.84 [3-51] | 18.82 ± 7.23 [5-31] | 18.63 ± 10.12 [3-40] |
| **N5** | 34.41 ± 14.95 [6-55] | 36.38 ± 17.06 [10-70] | 16.24 ± 6.03 [5-27] | 19.00 ± 9.79 [7-37] |
|  | **Sleep Onset Latency [min]** | |  |  |
|  | **BL** | **CL** |  |  |
| **N1** | 6 (4-8) [4-11] | 7 (6-8) [5-14] |  |  |
| **N2** | 7 (6-7) [6-8] | 6.5 (5-8) [2-11] |  |  |
| **N3** | 5 (4-8) [2-11] | 6 (4-7) [2-11] |  |  |
| **N4** | 4 (3-7) [0-11] | 6 (5-7) [4-9] |  |  |
| **N5** | 6 (5-7) [2-11] | 5 (4-5) [3-7] |  |  |

Sleep Onset Latency data show the median with lower and upper quartile in parentheses and minimum and maximum in square brackets; all other data show the mean ± standard deviation with minimum and maximum in square brackets; BL = bright light; CL = common lighting; min = minutes; N1-5 = night 1-5

# S4 HRV parameters

Table S6. Descriptive statistics of HRV parameters during the GNT

|  | **HR** | | **LF/HF** | |
| --- | --- | --- | --- | --- |
|  | **BL** | **CL** | **BL** | **CL** |
| **T1** | 72.28 ± 9.79 [55.17-86.69] | 81.84 ± 11.93 [60.14-108.01] | 0.37 ± 0.91 [-1.29-1.85] | 0.79 ± 0.91 [-0.51-2.95] |
| **T2** | 65.75 ± 8.33 [50.53-83.13] | 74.11 ± 9.92 [56.36-95.99] | 0.20 ± 0.85 [-0.96-1.69] | 0.64 ± 0.82 [-0.67-2.67] |
| **T3** | 64.90 ± 8.91 [49.97-86.66] | 73.12 ± 9.39 [52.89-90.97] | 0.37 ± 0.94 [-1.11-2.25] | 0.80 ± 0.66 [-0.08-2.45] |
| **T7** | 76.69 ± 10.60 [52.22-91.65] | 84.57 ± 10.34 [70.41-104.95] | 0.69 ± 0.87 [-1.16-1.75] | 0.93 ± 0.75 [-0.37-2.46] |
| **T8** | 66.40 ± 7.64 [49.15-79.52] | 72.80 ± 9.94 [60.41-99.95] | 0.43 ± 0.69 [-0.63-1.57] | 0.72 ± 0.89 [-0.86-2.91] |
| **T9** | 64.03 ± 7.05 [50.23-79.94] | 72.26 ± 10.16 [59.75-102.09] | 0.29 ± 0.80 [-1.06-1.95] | 0.53 ± 0.37 [-0.41-1.09] |
|  | **RMSSD** | | **SDNN** | |
|  | **BL** | **CL** | **BL** | **CL** |
| **T1** | 49.99 (35.12-57.82) [17.86-102.38] | 31.61 (21.77-41.93) [12.10-114.76] | 0.35 (0.00-1.02) [-1.29-1.85] | 0.79 (0.29-1.14) [-0.51-2.95] |
| **T2** | 61.08 (44.56-104.77) [25.05-158.87] | 34.21 (27.59-45.70) [19.80-138.37] | 0.03 (-0.39-0.75) [-0.96-1.69] | 0.32 (0.15-1.13) [-0.67-2.67] |
| **T3** | 71.31 (42.29-105.69) [20.52-201.45] | 37.89 (29.62-45.81) [23.95-103.84] | 0.28 (-0.22-0.89) [-1.11-2.25] | 0.68 (0.39-1.04) [-0.08-2.45] |
| **T7** | 38.36 (29.11-44.02) [13.74-102.82] | 34.34 (21.02-43.67) [13.74-119.31] | 0.70 (0.07-1.53) [-1.16-1.75] | 0.93 (0.51-1.41) [-0.37-2.46] |
| **T8** | 62.07 (42.11-77.87) [29.45-127.92] | 48.85 (33.59-58.03) [21.47-200.61] | 0.31 (-0.02-1.05) [-0.63-1.57] | 0.68 (0.23-0.93) [-0.86-2.91] |
| **T9** | 68.39 (41.56-91.79) [28.90-150.00] | 51.29 (44.18-62.31) [16.75-169.65] | 0.19 (0.05-0.84) [-1.06-1.95] | 0.53 (0.27-0.75) [-0.41-1.09] |

HR and LF/HF data show the mean ± standard deviation with minimum and maximum in square brackets; RMSSD and SDNN data show the median with lower and upper quartile in parentheses and minimum and maximum in square brackets; BL = bright light; CL = common lighting; T1-T9 = measurement time 1-9; HR = heart rate; LF/HF = ratio between LF and HF; LF = low frequency; HF = high frequency; RMSSD = square root of the mean squared differences of successive R-R intervals; SDNN = standard deviation of R-R intervals

Table S7. Descriptive statistics of HRV parameters during the 2-back task

|  | **HR** | | **LF/HF** | |
| --- | --- | --- | --- | --- |
|  | **BL** | **CL** | **BL** | **CL** |
| **T1** | 76.62 ± 9.34 [59.12-91.09] | 86.10 ± 10.61 [72.41-111.26] | 0.41 ± 0.70 [-1.01-1.37] | 0.98 ± 0.67 [-0.09-2.47] |
| **T2** | 67.27 ± 7.66 [52.30-84.28] | 77.05 ± 14.96 [55.07-120.19] | 0.23 ± 0.79 [-1.01-1.74] | 0.72 ± 0.77 [-0.20-2.29] |
| **T3** | 65.55 ± 9.18 [49.13-90.77] | 74.65 ± 10.90 [56.67-95.77] | 0.37 ± 0.79 [-1.35-1.53] | 0.78 ± 0.69 [-0.18-2.43] |
| **T7** | 80.17 ± 8.80 [57.38-95.36] | 87.30 ± 12.93 [68.76-112.89] | 0.87 ± 0.66 [-0.43-2.08] | 1.16 ± 0.85 [-0.42-2.59] |
| **T8** | 67.25 ± 6.99 [55.46-82.18] | 73.24 ± 10.95 [59.06-98.02] | 0.48 ± 0.86 [-1.61-2.13] | 0.61 ± 0.80 [-0.76-2.46] |
| **T9** | 66.24 ± 6.84 [52.76-83.84] | 72.53 ± 10.97 [58.44-105.79] | 0.45 ± 0.87 [-1.13-1.99] | 0.79 ± 0.55 [-0.44-1.75] |
|  | **RMSSD** | | **SDNN** | |
|  | **BL** | **CL** | **BL** | **CL** |
| **T1** | 45.32 (37.44-64.84) [20.94-105.55] | 49.70 (35.78-51.96) [24.26-130.47] | 36.79 (27.42-48.33) [17.12-106.01] | 31.21 (20.93-34.93) [16.37-108.60] |
| **T2** | 71.88 (56.02-87.89) [30.47-151.07] | 55.51 (46.21-59.51) [22.44-131.65] | 54.57 (47.48-85.61) [19.55-172.15] | 39.03 (27.56-47.71) [7.59-127.24] |
| **T3** | 62.05 (53.74-73.60) [25.77-121.84] | 49.54 (41.40-67.62) [30.34-108.31] | 54.67 (43.40-91.30) [14.40-142.71] | 35.41 (29.23-51.10) [18.93-109.42] |
| **T7** | 49.93 (37.84-67.39) [15.93-73.86] | 47.28 (33.38-58.25) [19.67-153.64] | 36.22 (26.41-43.95) [9.51-96.18] | 29.72 (16.15-44.19) [12.26-159.39] |
| **T8** | 65.86 (55.17-77.81) [34.76-127.84] | 56.34 (49.03-67.03) [38.35-156.63] | 55.34 (37.68-83.72) [31.13-104.48] | 44.92 (30.67-57.95) [26.02-165.86] |
| **T9** | 67.29 (51.48-88.87) [31.99-122.89] | 67.11 (50.18-83.99) [30.45-163.81] | 61.76 (38.09-85.60) [25.68-131.38] | 43.86 (40.00-53.49) [16.01-150.59] |

HR and LF/HF data show the mean ± standard deviation with minimum and maximum in square brackets; RMSSD and SDNN data show the median with lower and upper quartile in parentheses and minimum and maximum in square brackets; BL = bright light; CL = common lighting; T1-T9 = measurement time 1-9; HR = heart rate; LF/HF = ratio between LF and HF; LF = low frequency; HF = high frequency; RMSSD = square root of the mean squared differences of successive R-R intervals; SDNN = standard deviation of R-R intervals

Table S8. Descriptive statistics of HRV parameters during sleep

|  | **HR** | | **SDNN** | |
| --- | --- | --- | --- | --- |
|  | **BL** | **CL** | **BL** | **CL** |
| **Restful sleep** |  |  |  |  |
| N1 | 59.84 ± 6.41 [48.35-74.13] | 62.26 ± 6.94 [52.08-75.17] | 62.58 ± 16.99 [36.72-101.42] | 64.26 ± 27.52 [23.80-113.31] |
| N5 | 59.88 ± 4.75 [54.26-68.94] | 62.08 ± 6.62 [50.29-73.04] | 63.26 ± 15.21 [41.00-92.19] | 70.97 ± 29.49 [23.46-130.86] |
| **Restless sleep** |  |  |  |  |
| N1 | 65.34 ± 7.27 [50.77-78.15] | 69.01 ± 6.77 [57.96-79.91] | 101.26 ± 22.99 [52.81-135.21] | 95.92 ± 33.32 [36.66-157.48] |
| N5 | 67.29 ± 7.51 [58.05-89.21] | 68.74 ± 6.82 [54.74-78.69] | 94.39 ± 19.10 [58.04-132.97] | 98.13 ± 36.37 [41.72-186.23] |
|  | **RMSSD** | | **LF/HF** | |
|  | **BL** | **CL** | **BL** | **CL** |
| **Restful sleep** |  |  |  |  |
| N1 | 65.11 ± 27.71 [26.95-137.11] | 66.16 ± 39.20 [16.93-159.23] | -0.02 ± 0.76 [-1.37-1.68] | 0.08 ± 0.70 [-1.50-1.33] |
| N5 | 62.36 ± 20.83 [30.76-104.78] | 73.41 ± 40.71 [16.47-181.80] | 0.19 ± 0.71 [-1.02-1.61] | 0.13 ± 0.58 [-1.23-1.31] |
| **Restless sleep** |  |  |  |  |
| N1 | 65.61 ± 29.12 [22.82-135.18] | 62.41 ± 35.58 [13.65-145.93] | 0.80 ± 0.64 [-0.40-1.96] | 0.76 ± 0.59 [-0.47-1.54] |
| N5 | 56.90 ± 17.46 [31.84-92.94] | 67.39 ± 36.81 [17.46-167.44] | 0.93 ± 0.63 [-0.01-2.34] | 0.71 ± 0.43 [-0.26-1.47] |

Data show the mean ± standard deviation with minimum and maximum in square brackets; BL = bright light; CL = common lighting; N1 = Night 1; N5 = Night 5; HR = heart rate; SDNN = standard deviation of R-R intervals; RMSSD = square root of the mean squared differences of successive R-R intervals; LF = low frequency; HF = high frequency; LF/HF = ratio between LF and HF

Table S9. Summary of ANOVA results (p-values) of HRV parameters during cognitive performance tasks

|  | **2-back** | | | | | | |
| --- | --- | --- | --- | --- | --- | --- | --- |
| **Parameter** | **Group** | **Day** | **Day * Group** | **Measurement** | **Measurement * Group** | **Day * Measurement** | **Day * Measurement * Group** |
| HR | **.009** | .932 | .148 | **< .001** | .900 | .101 | .872 |
| LF/HF | .083 | .096 | .176 | **< .001** | .674 | .106 | .605 |
|  | **GNT** | | | | | | |
| **Parameter** | **Group** | **Day** | **Day * Group** | **Measurement** | **Measurement * Group** | **Day * Measurement** | **Day * Measurement * Group** |
| HR | **.007** | .456 | .569 | **< .001** | .622 | **.007** | .615 |
| LF/HF | .150 | .437 | .338 | **.009** | .967 | **.003** | .983 |

GNT = go-/no-go task; Day = first day vs. fifth day; Measurement = measurement time (HRV was measured during T1-T3 and T7-T9); HR = heart rate; LF/HF = ratio between LF and HF; LF = low frequency; HF = high frequency. Significant results are marked in bold text.

Table S10. Summary of Friedman tests (measurement) and Mann-Whitney U tests (T1-T9) of HRV parameters during cognitive performance tasks (p-values)

|  | **2-back** | | | | | | |
| --- | --- | --- | --- | --- | --- | --- | --- |
| **Parameter** | **Measurement** | **T1** | **T2** | **T3** | **T7** | **T8** | **T9** |
| SDNN | **< .001** | .709 | .034 | .025 | .662 | .834 | .984 |
| RMSSD | **< .001** | .151 | **.015** | **.017** | .458 | .303 | .347 |
|  | **GNT** | | | | | | |
| **Parameter** | **Measurement** | **T1** | **T2** | **T3** | **T7** | **T8** | **T9** |
| SDNN | **< .001** | .382 | .022 | .029 | .364 | .684 | .779 |
| RMSSD | **< .001** | .072 | **.004** | **.006** | .171 | .072 | .298 |

GNT = go-/no-go taskSDNN = standard deviation of R-R intervals; RMSSD = square root of the mean squared differences of successive R-R intervals. Significant results are marked in bold text.

## **S4.1 Physiological stress markers during cognitive performance tasks**

Descriptive statistics of HRV parameters during the cognitive performance tasks are presented in Table S6 and Table S7. ANOVA results are summarized in Table S9, results for non-parametric analyses are summarized in Table S10.

Regarding heart rate, we found a significant main effect of group during the 2-back F(1, 33) = 7.59, p = .009, η_p_² = .187) as well as the GNT (F(1, 33) = 8.30, p = .007, η_p_² = .201) with lower average heart rate in the BL group.

Further, we found highly significant effects of measurement time in heart rate and LF/HF independent of the experimental condition. Both parameters improved significantly (i.e. indicated lower stress) in both groups. This phenomenon was present on day 1 as well as day 5. Similar effects were also observed in the interaction of day and measurement time in both parameters, but only during the GNT.

Non-parametric analyses of SDNN and RMSSD also revealed highly significant effects of measurement time (see Table S10). In RMSSD data, however, Mann-Whitney U tests further revealed significant differences between groups during both cognitive performance tasks on the first day at T2 (2-back: U = 80.00, p = .015, r = -.477; GNT: U = 67.00, p = .004, r = -.562) and T3 (2-back: U = 81.00, p = .017, r = -.471; GNT: U = 71.00, p = .006, r = -.536), but not on the fifth day at T8 (2-back: U = 121.00, p = .303, r = -.209; GNT: 98.00, p = .072, r = -.359) or T9 (2-back: U = 124.00, p = .347, r = -.190; GNT: U = 121.00, p = .298; r = -.209).

During the GNT, RMSSD increased significantly in the BL group from T1 to T2 (z = -2.77, p = .004, r = -.743) and T1 to T3 (z = -2.72, p = .005, r = -.731), but not T2 to T3 (z = -0.63, p = .551, r = -.170). In the CL group, RMSSD did not change significantly (T1-T2: z = -1.54, p = .132, r = -.425; T2-T3: z = -1.87, p = .064, r = -.516; T1-T3: z = -1.35, p = .190, r = -.373). On the fifth day (i.e. T7-T9), RMSSD increased in both groups similarly: In the CL group, T7 to T8 (z = -3.48, p < .001, r = -.961) and T7 to T9 (z = -3.48, p < .001, r = -.961) increased significantly, but T8 to T9 did not (z = -1.44, p = .159, r = -.399). In the BL group, T7 to T8 (z = -3.68, p < .001, r = -.988) and T7 to T9 (z = -3.72, p < .001, r = -1.000) also increased significantly, but T8 to T9 did not (z = -1.89, p = .060, r = -.509).

During the 2-back, RMSSD increased significantly in the CL group from T1 to T2 (z = -2.30, p = .020, r = -.634), but not from T2 to T3 (z = 0.17, p = .890, r = .046) or T1 to T3 (z = -1.92, p = .057, r = -.529), while in the BL group, RMSSD also increased significantly from T1 to T2 (z = -3.29, p < .001, r = -.883) and T1 to T3 (z = -3.46, p < .001, r = -.930), but not from T2 to T3 (z = -0.07, p = .966, r = -.018). On the fifth day, both groups again showed similar results: In the CL group, T7 to T8 (z = -3.10, p < .001, r = -.856) and T7 to T9 (z = -2.86, p = .003, r = -.791) increased significantly, but T8 to T9 did not (z = -0.40, p = .712, r = .111). In the BL group, T7 to T8 (z = -3.51, p < .001, r = -.942) and T7 to T9 (z = -3.68, p < .001, r = -.988) also increased significantly, but T8 to T9 did not (z = -1.63, p = .108, r = -.439).

# S5 Photometric documentation

Table S11. Measured illuminances and CCTs at eye level

|  | **CCT (K)** | **Illuminance (lx)** | **Melanopic EDI (lx)** | **S-cone-opic EDI (lx)** | **M-cone-opic EDI (lx)** | **L-cone-opic EDI (lx)** | **Rhodopic EDI (lx)** |
| --- | --- | --- | --- | --- | --- | --- | --- |
| **BL** |  |  |  |  |  |  |  |
| Workplace 1 | 3813 | 1515 | 987 | 908 | 1324 | 1523 | 1077 |
| Workplace 2 | 3881 | 1546 | 1030 | 947 | 1357 | 1553 | 1118 |
| Workplace 3 | 3862 | 1552 | 1040 | 963 | 1362 | 1562 | 1124 |
| Workplace 4 | 3917 | 1509 | 1010 | 944 | 1327 | 1516 | 1095 |
| **CL** |  |  |  |  |  |  |  |
| Workplace 1 | 2708 | 92 | 45 | 33 | 72 | 93 | 54 |
| Workplace 2 | 2845 | 92 | 49 | 36 | 74 | 94 | 57 |
| Workplace 3 | 2980 | 101 | 57 | 44 | 83 | 103 | 65 |
| Workplace 4 | 2819 | 93 | 48 | 35 | 74 | 94 | 56 |

BL = bright light; CL = control group; CCT = correlated color temperature; EDI = equivalent daylight illuminance

Fig S1. Spectral distribution of all four workplaces in the BL condition


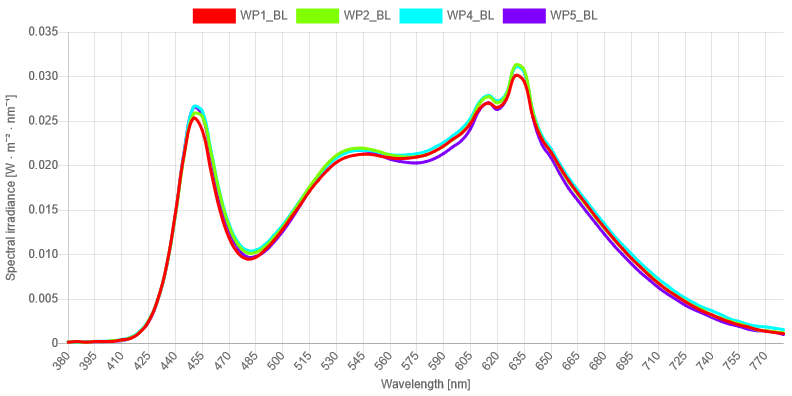


Fig S2. Spectral distribution of all four workplaces in the CL condition


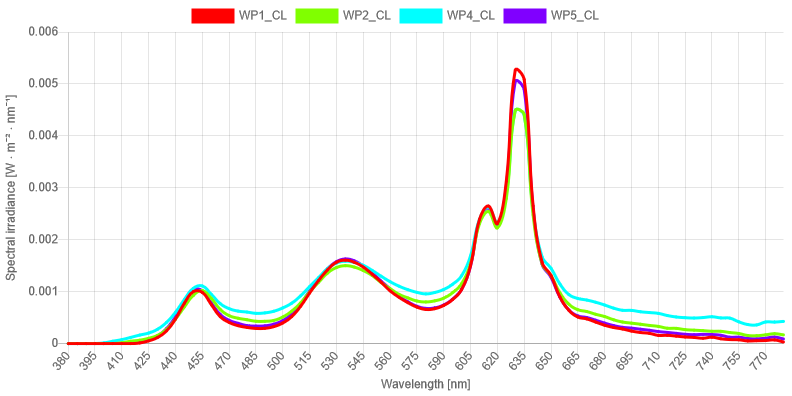

Supplement: Supporting Information 4 — In the .docx file “ReducingExamAnxiety_SupportingInformation,” Tables S1–S11, Figures S1 and S2, and the results regarding physiological stress markers during cognitive performance tasks are available. [file 1422406.f4.docx]
